# Decode-seq: a practical approach to improve differential gene expression analysis

**DOI:** 10.1186/s13059-020-01966-9

**Published:** 2020-03-23

**Authors:** Yingshu Li, Hang Yang, Hujun Zhang, Yongjie Liu, Hanqiao Shang, Herong Zhao, Ting Zhang, Qiang Tu

**Affiliations:** 1 State Key Laboratory of Molecular Developmental Biology, Institute of Genetics and Developmental Biology, Innovation Academy for Seed Design, Chinese Academy of Sciences, Beijing, 100101 China; 2 Key Laboratory of Genetic Network Biology, Institute of Genetics and Developmental Biology, Chinese Academy of Sciences, Beijing, 100101 China; 3 University of Chinese Academy of Sciences, Beijing, 100049 China

**Keywords:** Differential expression, RNA-seq, Replication, Medaka, Germ cell

## Abstract

Many differential gene expression analyses are conducted with an inadequate number of biological replicates. We describe an easy and effective RNA-seq approach using molecular barcoding to enable profiling of a large number of replicates simultaneously. This approach significantly improves the performance of differential gene expression analysis. Using this approach in medaka (*Oryzias latipes*), we discover novel genes with sexually dimorphic expression and genes necessary for germ cell development. Our results also demonstrate why the common practice of using only three replicates in differential gene expression analysis should be abandoned.

Identification of differentially expressed genes (DEGs) is a fundamental step for many biomedical studies. The advent of RNA-sequencing technology ushered in a new era for DEG discovery. Single-cell RNA-sequencing has emerged as a powerful tool to achieve this end. However, due to the technical difficulties and the high cost associated with single-cell technologies, bulk RNA-sequencing remains the most popular approach for differential expression (DE) analysis in many biomedical fields. For example, 2 widely used algorithms for DE analysis, edgeR [1] and DESeq2 [2], have been cited over 10,000 times.

Analysis with inadequate replicates affects the accuracy and has the potential to invalidate many conclusions arrived at in such a manner. Biostatisticians have long encouraged the use of more biological replicates to achieve sufficient statistical power to call DEG correctly [3–6]. Nevertheless, many biologists are unaware of the appropriate number of replicates required to reach a threshold level of statistical power. We roughly estimated the replicate number commonly employed in publicly released studies. From NCBI GEO (Gene Expression Omnibus) database, we extracted 1,167 expression profiling studies which cited edgeR or DESeq2 and named the associated samples like “rep1” or “replicate 1” etc., which enabled us to estimate the replicate number used. From these studies, we found 39% of studies using only two replicates, 43% using three replicates, and only 18% using four or more replicates. The median replicate number was 3 (Additional file 1: Fig. S1). At this level of replication, only the most strongly changing gene can be identified. Our approximate estimation showed that presently most studies are underpowered to detect significant differences at a biologically meaningful level. Considering the wide application of DE analysis, the number of underpowered studies is surprisingly large.

This fundamental problem could be due to limitations of current bulk RNA-sequencing profiling methods, including relatively high cost and complex processing, which prevents researchers from using adequate replicates. For example, the cost and time needed to profile several dozens of samples would impose significant cost constraints on most research projects. Another scenario is that samples to be profiled are diminutive. To meet the requirement of the quantity of input RNA for the sequencing, researchers have to pool multiple samples together, which constrains the usable sample number even further. Therefore, the lack of practical experimental approaches prevents biologists from employing adequate replicates to achieve sufficient power in DEG discovery studies.

DNA barcode technology provides a solution to the problem. The capacities of current sequencing platforms greatly exceed the requirements of most single experiments. Therefore, various strategies of library multiplexing have been developed. For example, with Illumina TruSeq RNA technology, each library is processed individually and tagged with a short DNA barcode in a late step (ligate adapters), and a few dozens of libraries could be sequenced simultaneously in a single run. Most RNA-seq technologies are compatible with this late multiplexing strategy.

In recent years, single-cell RNA-seq (scRNA-seq) technologies have been developed rapidly. Some full-length scRNA-seq technologies (e.g., Smart-seq2 [7]) process each cell and tag each library individually in a late step (library amplification). Many other scRNA-seq technologies (e.g., STRT-seq [8], MARS-seq [9], Drop-seq [10], Cel-seq2 [11], Seq-well [12], Microwell-seq [13], and SPLiT-seq [14]) add barcodes from the early step (reverse transcription), then pool all samples together for the downstream processing. Since the barcodes are added before fragmentation, only one end of the transcript can be tagged. Thus, this early multiplexing strategy sacrifices the information of full-length transcripts while it enormously minimizes the library construction labor and cost and increases the multiplexity to the level of thousands or even more.

In addition to the use of DNA barcode to identify different samples/cells, another type of barcode termed as the unique molecular identifier (UMI) was also developed [15, 16]. A short random sequence is added to each cDNA molecule during reverse transcription, and quantification of transcripts is achieved by counting the number of distinct UMIs instead of by counting the number of reads. This method significantly reduced the inherent noise introduced by the PCR amplification and improved the accuracy of quantification.

A few methods have adopted these barcoding technologies to bulk RNA-seq samples. SCRB-seq [17] was originally developed for single-cell analysis, which adds both cell barcodes and UMIs to cDNAs by early multiplexing, then specifically enriches 3^′^ fragments for gene expression quantification. This method was used in bulk RNA-seq analysis in several studies and then was further optimized to form an approach called BRB-seq [18]. By 3^′^ end barcoding and enrichment, BRB-seq is able to produce dozens of libraries at a very low cost, which showed great potential for DE analysis.

The major problem of BRB-seq is that its library structure makes the sequencing difficult. Nucleotide diversity is important for the generation of high-quality data. BRB-seq libraries contain a poly(T) stretch introduced by the reverse transcription primer. Sequencing through such a low diversity region typically yields poor read qualities [19]. There are three ways to deal with this problem. The first is to adjust the read length to avoid the poly(T) stretch. The BRB-seq study used this way, and the read1 sequencing was set to 6–21 cycles. However, this adjustment will affect the entire flow cell, which means that users have to devote the entire flow cell for BRB-seq libraries. The sequencing cost of a flow cell is prohibitive for most users. The second solution is to add the PhiX control library as spike-in to provide enough sequence complexity to cover the poly(T) stretch. However, BRB-seq uses a customized read1 primer which cannot sequence the PhiX control and other standard Illumina libraries. The third solution is simply devoting the entire lane for BRB-seq libraries without the PhiX control and bearing the low quality of reads. In short, although BRB-seq showed its high multiplexing capacity to reduce the library construction cost, it is technically challenging or expensive to sequence the libraries. To our knowledge, there is currently still no practical approach to achieve early multiplexing for bulk RNA-seq with low cost of both library construction and sequencing.

To address this issue, here, we describe a practical approach Decode-seq (Differential Expression analysis by barCODEd SEQuencing) to overcome the current limitations and enable more accurate DEG discovery. This approach is technically simple, is compatible with standard sequencing settings, and significantly reduces the cost of both library construction and sequencing. With the high number of replicates, it dramatically improved sensitivity and reduced the false discovery rate. We applied this method in analyzing medaka fish at the early stage of sex determination and discovered multiple novel sexually dimorphically expressed genes, some of which are required for germ cell development.

Decode-seq adopts both sample barcode and molecular barcode in bulk RNA-seq for the DEG discovery purpose. During the reverse transcription, a fragment containing a unique sample identifier (USI) and a unique molecule identifier (UMI) is added to the 3^′^ end of the first-strand cDNA corresponding to the 5^′^ end of the transcript using the template switch method (Additional file 1: Fig. S2). With these identifiers, a large number of samples can be mixed into one pool and processed for library construction. After the tagmentation step, the fragments containing UMI, USI, and 5^′^ end of the transcript are enrich by PCR and sequenced using the Illumina platform. Thus, USIs carry the sample identification and enable multiplexing; UMIs carry the origin cDNA molecule identification and enable amplification of a small amount of starting material without severe quantification bias; 5^′^ end sequencing avoid the poly(T) stretch of the reverse transcription primer which causes base calling difficulty. In addition, 5^′^ end instead of full-length sequencing also reduce the number of reads needed. The Decode-seq libraries can be sequenced with standard Illumina sequencing primers. Therefore, it can be sequenced together with any common libraries, without the requirement of dedicating the entire lane or flow cell. Altogether, these designs significantly reduce the time and cost required for profiling a large number of samples. For example, in this study, 30 samples were profiled in 1 Decode-seq library, which reduced the library construction cost to about 5%; sequencing depth was also reduced to about 10–20% (6M vs. 30–60M); in total, the cost of library preparation and sequencing is reduce to about 10–15%.

We first evaluated the performance of Decode-seq using human/mouse RNA mixtures of known fold change. Sample mix5 was human RNA containing 5% mouse RNA, while mix1 was human RNA containing 1% mouse RNA. Thus, the difference of mouse genes between two sample mixes was fivefold. Each mix was profiled 30 times at the 100 ng level. Reads were processed by customized scripts (Supplementary Data). DE analysis was performed with edgeR. As we knew all mouse genes were true DEGs and all human genes were not, we can calculate sensitivity, specificity, and all other performance statistics of DE callings with different numbers of replicates. For the statistics to be comparable, we fixed the specificity (percentage of human genes which were called non-DE) at the same level (95%), then calculated the sensitivity (percentage of mouse genes which were called DE) and false discovery rate (percentage of human genes in all identified DEGs) with replicates downsampled from 30 to 2 pairs. As illustrated in a calculation using 3 pairs of replicate (Fig. 1a), the sensitivity is 31.0% as only 1372 (red dots) out of 4432 mouse genes (1372 red and 3060 orange dots) were identified as DEGs, while the false discovery rate was as high as 33.8%, as out of 2072 recognized DEGs (1372 red and 700 blue dots), 700 were human genes (blue dots). When the replicate number increased to 30, measurements of human and mouse genes were clearly separated (Fig. 1b). The sensitivity dramatically increased to 95.1%, and the false discovery rate dropped to 14.2%. The calculation was repeated 100 times for each number of replicates, and the results clearly showed the trends that the performance was greatly improved when more replicates were used (Fig. 1c). This conclusion is consistent with previous studies [3–6].

**Fig. 1:**
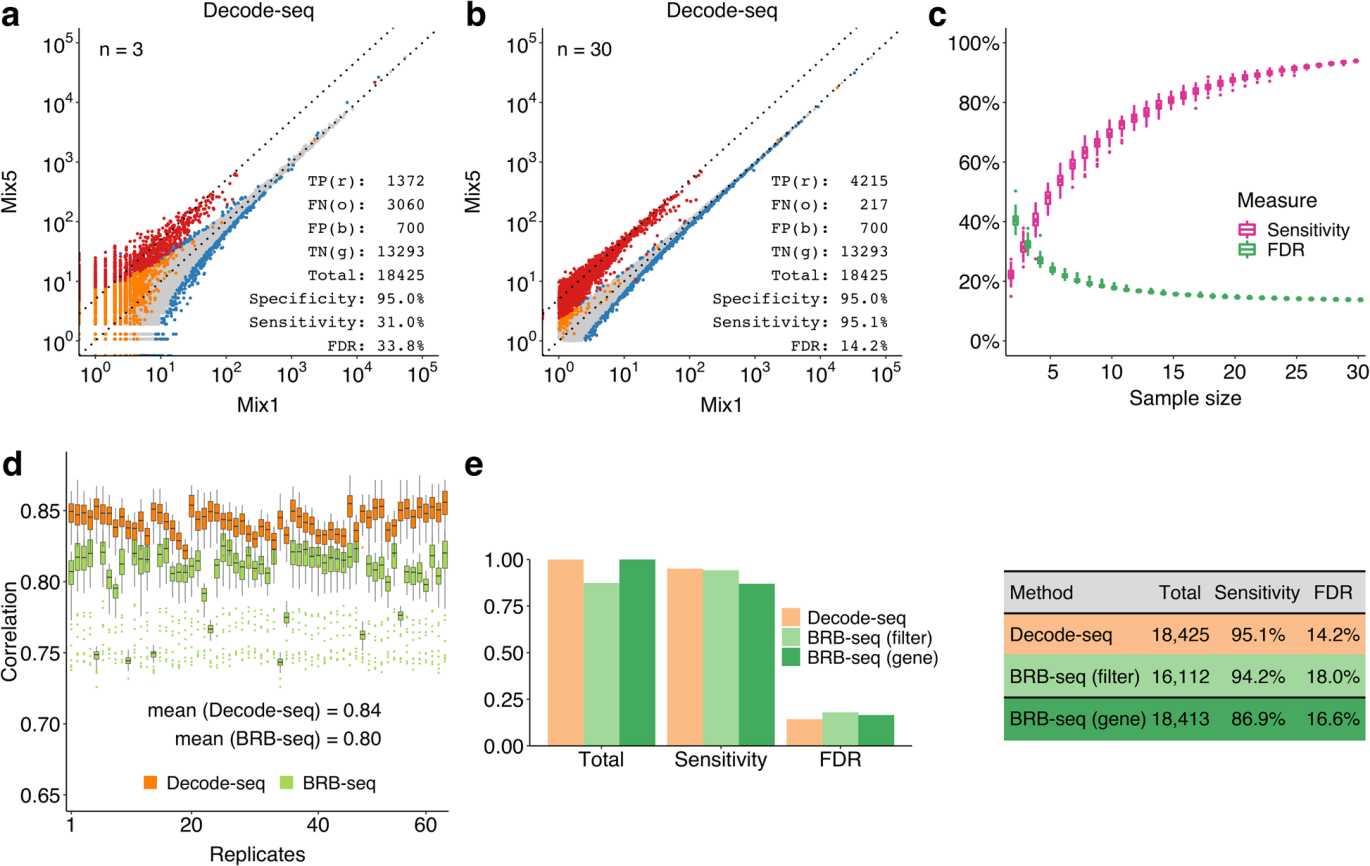
Performance evaluation of Decode-seq. **a** Differential expression analysis with 3 pairs of replicates. True positive (TP, red dots), mouse genes which were called DE; false negative (FN, orange dots), mouse genes which were called non-DE; true negative (TN, gray dots), human genes which were called non-DE; false positive (FP, blue dots), human genes which were called DE. Specificity = TN/(TN + FP), and it was fixed to 95% in all calculation. Sensitivity = TP/(TP + FN). False discovery rate = FP/(TP + FP). **b** DE analysis with 30 pairs of replicates. The sensitivity increased to 95.1%, and the false discovery rate dropped to 14.2%. **c** DE performance related to replicate number calculated by random downsampling of 30-pair data. Each replicate number was calculated 100 times. Sensitivity and false discovery rate were improved dramatically when the number of replicates increased. **d** Spearman’s correlations among replicates of Decode-seq and BRB-seq. Each replicate was compared with all other replicates of Decode-seq and BRB-seq, respectively. The distribution of these correlations was shown as the box for each replicate. In the Decode-seq group, replicate has higher correlations with each other, indicating the higher reproducibility. **e** DE performance of Decode-seq and BRB-seq. Bars in three colors represent DE performance of three sets: Decode-seq, BRB-seq with the same filter parameters as Decode-seq, and BRB-seq with the same total gene as Decode-seq. When using the same filter parameters, BRB-seq detected fewer genes. When using loose filter parameters to ensure the same total gene, BRB-seq gave a lower sensitivity

We downsampled the sequencing depth and found that 5.9M reads per sample was good for the purpose of this experiment (Additional file 1: Fig. S3). The sequencing depth should be determined on different purposes, for example, if detection of rare transcripts is required, more reads are needed to ensure the saturated UMI counts. We also tested this approach in threefold difference scenario (Additional file 1: Fig. S4) or lower input scenario (10 ng and 1 ng level) (Additional file 1: Fig. S5). It consistently improved the DE performance in all situations. In brief, Decode-seq is able to profile 30 pairs of biological replicates thus dramatically improved the DE performance at much less cost.

We also compared the performance of Decode-seq and BRB-seq. As discussed above, the structure of BRB-seq library is not compatible with standard Illumina sequencing conditions. Users have to either devote the entire flow cell and adjust the sequencing cycles to ensure high-quality reads, or devote one lane and use the customized primer but bear the low-quality reads. The BRB-seq study used the former strategy. Due to the prohibitive cost of using an entire flow cell, we did not compare two methods this way. Instead, we used the latter strategy, which cannot generate high-quality reads but is more practical for most labs. As expected, the read quality was adversely affected by the poly(T) repeats (Additional file 1: Fig. S6). Correspondingly, the mapping rate was only 65% while that of Decode-seq was 80%. We calculated the Spearman’s correlation coefficients between each replicate and all other replicates in the groups of Decode-seq and BRB-seq, respectively (Fig. 1d, Additional file 1: Fig. S7). The result showed that the correlations among BRB-seq replicates were lower than those of Decode-seq, indicating a lower reproducibility. In the comparison of DE performance (Fig. 1e, Additional file 1: Fig. S8), when the same filter parameters of edgeR were used, BRB-seq detected fewer genes than Decode-seq (16,112 vs. 18,425). If we loosed the filter parameters to ensure the same total number of genes detected, BRB-seq showed lower sensitivity (86.9% vs. 95.1%). In short, with standard sequencing settings, BRB-seq generated reads with poor quality, which further reduced the performance of DE analysis. Decode-seq uses standard sequencing primers and contains no low diversity poly(T) repeats. Therefore, users do not have to devote the entire flow cell or lane, which significantly reduced the sequencing cost and make the experiment design flexible. In fact, we routinely sequenced the Decode-seq libraries with only 10% lane per library for the quality control purpose. However, Decode-seq is 5^′^ based and uses the template switching mechanism which biases towards full-length cDNA molecules. BRB-seq is 3^′^ based and uses the nick translation mechanism. Therefore, if these are the concerns and customized sequencing is not an issue, BRB-seq is an attractive alternative solution to achieve multiplexing.

Next, to test the applicability of Decode-seq, we sought to apply Decode-seq to address a real biological question. Sex determination is an essential step in the fate decisions of germ cells. Medaka (*Oryzias latipes*), a small egg-laying freshwater teleost, is an important model organism for studying vertebrate sex determination [20]. Several key factors controlling medaka sex determination are known, including *DMY* [21], *gsdf* [22], *foxl2* [23], and *foxl3* [24]. However, the cohort of genes involved in this process is largely unknown. It was reported that the first appearance of morphological sex difference in medaka is the difference in the number of germ cells, as these cells in female undergo a rapid proliferation while they remain quiescent in male. At stage 39 (just hatching), the average numbers of germ cells in male and female are about 80 and 120 [25]. Therefore, DEGs at this stage are expressed differentially in only a few hundred cells (germ cells and somatic gonadal cells) per fish. It is a great challenge to correctly identify DEGs between male and female at this stage. Using Decode-seq, we profiled gene expression of 30 male fry and 30 female fry (Fig. 2a). Top 300 highly sexually dimorphically expressed genes ranked by adjusted *p* values were selected for further analysis (Additional file 2: Table S1). One hundred twenty-one genes in male and 179 genes in female were identified as DEGs, including *gsdf* and *foxl3*. Some known markers (e.g., *DMY*, *foxl2*) are expressed at low levels. Although these genes showed clear dimorphic expression in the data, they do not fall into the top 300 list. We selected 25 genes based on the expression, gene structure, and functional clues from this top 300 list for further study. In the qPCR validation (Fig. 2b), 20 (80%) of them were confirmed to be significantly differentially expressed. We did the downsampling analysis similar to the human/mouse RNA experiment, and the conclusion was the same: much fewer genes could be identified with fewer replicates (Fig. 2c). Further, we used *in situ* hybridization chain reaction (HCR) [26] to check the cellular location of *cd74a*, a novel DEG (Fig. 2d). The expression of this gene co-localized with the germ cell marker *vasa*, instead of the somatic cell marker *sox9b*. This indicates *cd74a* is specifically expressed in germ cells. The dimorphic expression pattern of *cd74a* is consistent with the dimorphic different number of germ cells. In fact, Decode-seq also identified the germ cell marker *vasa* as a DEG in the top 300 list. Lastly, we knocked out a novel DEG (ENSORLG00000007290) using a rapid knockout method to generate F0 mutants [27, 28]. The mutant showed severe germ cell depletion (Fig. 2e–h, Additional file 1: Fig. S9), which indicates that this gene is required for germ cell development. Briefly, Decode-seq was able to identify novel genes with differential expression and potential key functions in very challenging scenarios.

**Fig. 2:**
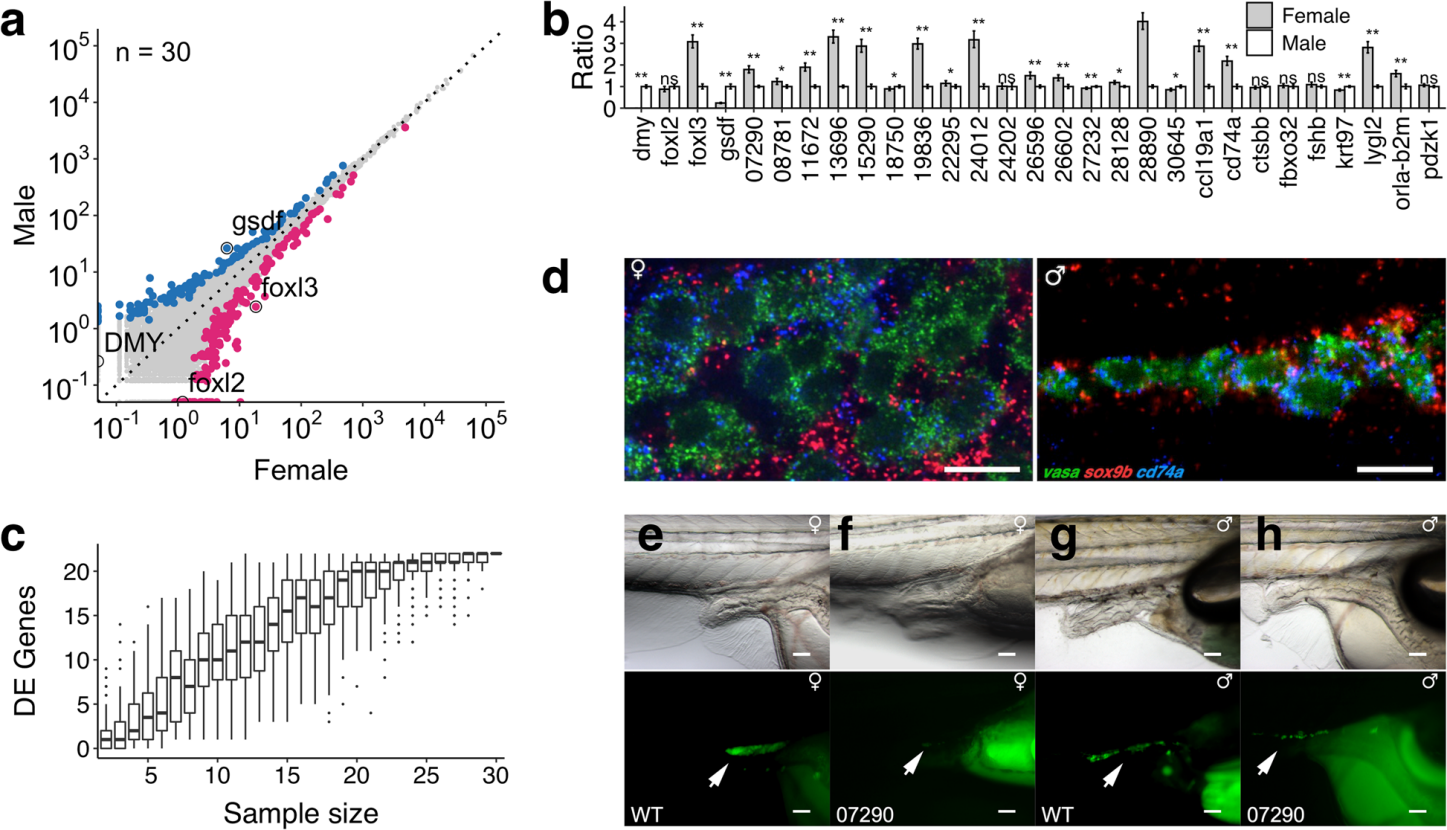
Differential gene expression analysis of sex determination of medaka. **a** Expression profiling of 30 pairs of medaka male/female fry with Decode-seq. Top 300 genes were color coded. Blue dots, genes expressed higher in male; red dots, genes expressed higher in female; black circled dots, known genes with dimorphic expression. **b** qPCR validation of identified DEGs, including 4 known markers and 25 novel DEGs. Error bar: SE. *p < 0.05, **p < 0.01. **c** Sample size downsampling shows much fewer DEGs would be identified with fewer replicates. **d** Hybridization chain reaction validation of *cd74a*. Green signal: *vasa*, expressed in germ cells; red signal: *sox9b*, expressed in somatic cells; blue signal: *cd74a*, expressed in germ cells. Scale bar: 10 *μ* m. **e**–**h** Functional validation of ENSORLG00000007290 using genetic knockout. Medaka fry at stage 39 (hatching) was used. Arrows indicate the GFP-labeled germ cells. Scale bar, 100 *μ* m. **e** Wild-type female fry, a cluster of germ cells with strong GFP signal are visible. **f** Mutant female fry, germ cells are barely visible. **g** Wild-type male fry, a cluster of germ cells are visible. **h** Mutant male fry, germ cells are largely depleted

The salient features of Decode-seq are as follows: (1) multiplexing—a large number of samples can be mixed in one library, which significantly reduces the time and cost of library preparation; (2) high sensitivity—trace amounts of RNA can be profiled, and therefore, small tissue samples can be analyzed without being pooled together; (3) 5^′^ cDNA end enrichment—this greatly reduces sequencing depth required compared to the full-length sequencing and overcomes the difficulty of 3^′^ end sequencing; and (4) compatible with standard Illumina sequencing settings—it does not require customized sequencing cycle numbers or primers, so users do not have to devote the entire flow cell or lane for sequencing. This feature significantly reduces sequencing cost. Given the high performance and low cost associated with this approach, we anticipate Decode-seq to be widely used to improve differential gene expression analysis in many instances where, for one reason or another, the single-cell analysis is not possible or affordable. Despite biostatisticians’ longtime appeal of using more replicates to achieve sufficient statistical power, it is still very common to employ only two or three replicates. Here, we described an easy and effective experimental approach for DEG discovery. Depending on the purpose of studies and resources available, researchers could use either 5^′^-based Decode-seq or 3^′^-based BRB-seq to achieve multiplexing. Unless samples are precious, full-length sequencing is necessary, or underpower is not an issue, there are few reasons to use inadequate replicates. Therefore, we appeal that the common practice of using three replicates in differential gene expression analysis should be avoided if possible.

## Supplementary information

**Additional file 1** Supplementary figures. **Fig S1**: Distribution of replicate numbers employed in surveyed GEO studies. **Fig S2**: Design of Decode-seq. **Fig S3**: Downsampling of sequencing depth. **Fig S4**: Performance evaluation of Decode-seq at 3-fold change level using human/mouse RNA mixes. **Fig S5**: Performance evaluation of Decode-seq with 10 ng and 1 ng RNA at 5-fold change level. **Fig S6**: Sequencing quality scores and nucleotide distribution of BRB-seq and Decode-seq. **Fig S7**: Spearman’s correlations of human gene UMI counts between technical replicates of Decode-seq and BRB-seq. **Fig S8**: DE performance of BRB-seq and Decode-seq when using different edgeR filtering parameters. **Fig S9**: Genetic knockout of ENSORLG00000007290 by four-guide Cas9 RNP.

**Additional file 2** Supplementary tables. **Table S1**: Top 300 genes with sexually dimorphic expression identified in this study. **Table S2**: Primers and gRNAs used in this study. **Table S3**: Sequencing stats of all libraries sequenced in this study.

**Additional file 3** Supplementary methods.

**Additional file 4** Supplementary data: the experimental protocol and computational analysis pipeline for decode-seq.

**Additional file 5** Review history.

## Data Availability

Detailed methods used in this study are described in Additional file 3: Supplementary Methods. Raw sequencing reads and processed count tables are available in NCBI Gene Expression Omnibus database under accession GSE130014 [30]. The experimental protocol and computational analysis pipeline of Decode-seq are included as Additional file 4: Supplementary Data, and also available on GitHub [31] (licensed under GNU General Public License v3.0), Zenodo [32], and our lab website [33].
