# Supplementary material for: Decode-seq: a practical approach to improve differential gene expression analysis
Source: Genome Biol. 2020 Mar 23;21:66. doi: 10.1186/s13059-020-01966-9 (PMC7087377; doi:10.1186/s13059-020-01966-9)
Supplement: Supplementary file 1 — Additional file 1 Supplementary figures. Fig S1: Distribution of replicate numbers employed in surveyed GEO studies. Fig S2: Design of Decode-seq. Fig S3: Downsampling of sequencing depth. Fig S4: Performance evaluation of Decode-seq at 3-fold change level using human/mouse RNA mixes. Fig S5: Performance evaluation of Decode-seq with 10 ng and 1 ng RNA at 5-fold change level. Fig S6: Sequencing quality scores and nucleotide distribution of BRB-seq and Decode-seq. Fig S7: Spearman’s correlations of human gene UMI counts between technical replicates of Decode-seq and BRB-seq. Fig S8: DE performance of BRB-seq and Decode-seq when using different edgeR filtering parameters. Fig S9: Genetic knockout of ENSORLG00000007290 by four-guide Cas9 RNP. [file 13059_2020_1966_MOESM1_ESM.pdf]

Decode-seq: a practical approach to improve differential gene expression analysis

Supplementary Figures

Yingshu Li[1,2,3], Hang Yang[1,2,3], Hujun Zhang[1,2,3], Yongjie Liu[1,2,3], Hanqiao Shang[1,2], Herong Zhao[1,2,3], Ting Zhang[1,2], and Qiang Tu[1,2,3,*]

[1] *State Key Laboratory for Molecular and Developmental Biology, Institute of Genetics and Developmental Biology, Innovation Academy for Seed Design, Chinese Academy of Sciences, Beijing 100101, China*
[2] *Key Laboratory of Genetic Network Biology, Institute of Genetics and Developmental Biology, Chinese Academy of Sciences, Beijing 100101, China.*
[3] *University of Chinese Academy of Sciences, Beijing 100049, China*

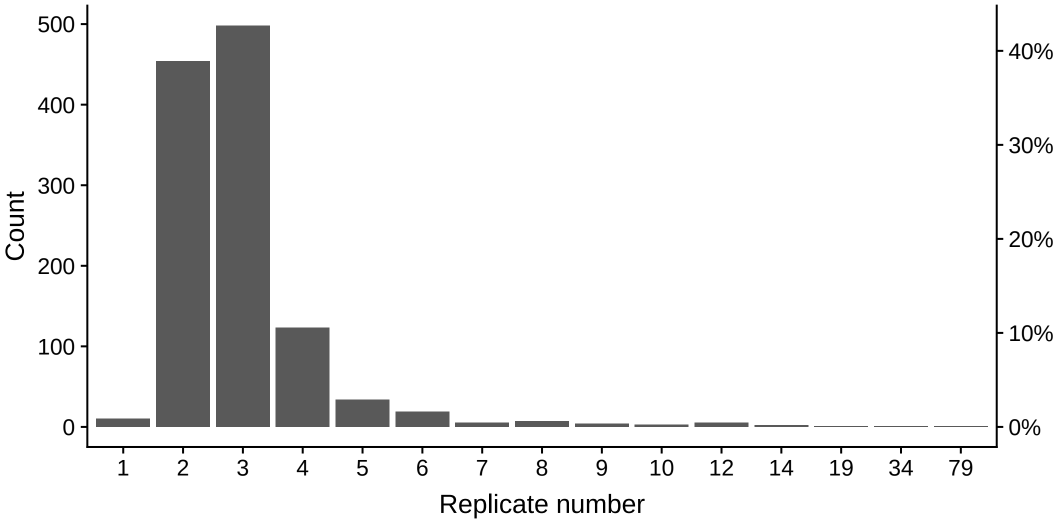

Fig S1: Distribution of replicate numbers employed in surveyed GEO studies. From NCBI GEO (Gene Expression Omnibus) database, we extracted 1,167 expression profiling studies which cited edgeR or DESeq2 and named the associated samples like 'rep1' or 'replicate 1' etc. We used these sample names to estimate the replicate numbers employed in these studies. Among them, 39% used only 2 replicates, 43% used 3 replicates, while only 18% used 4 or more replicates.

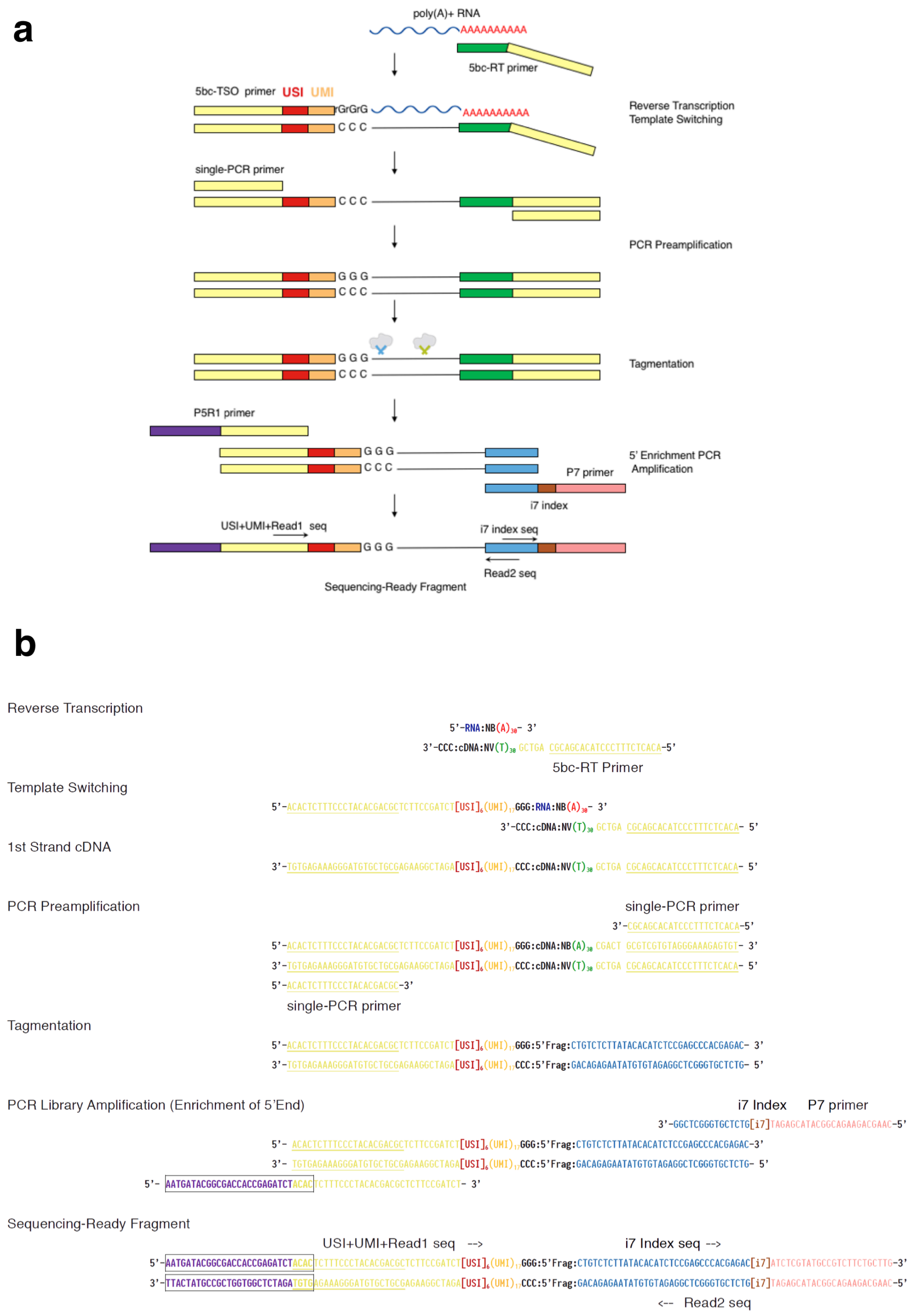

Fig S2: Design of Decode-seq. (a) Overview of Decode-seq workflow. (b) Detailed sequences used in each step.

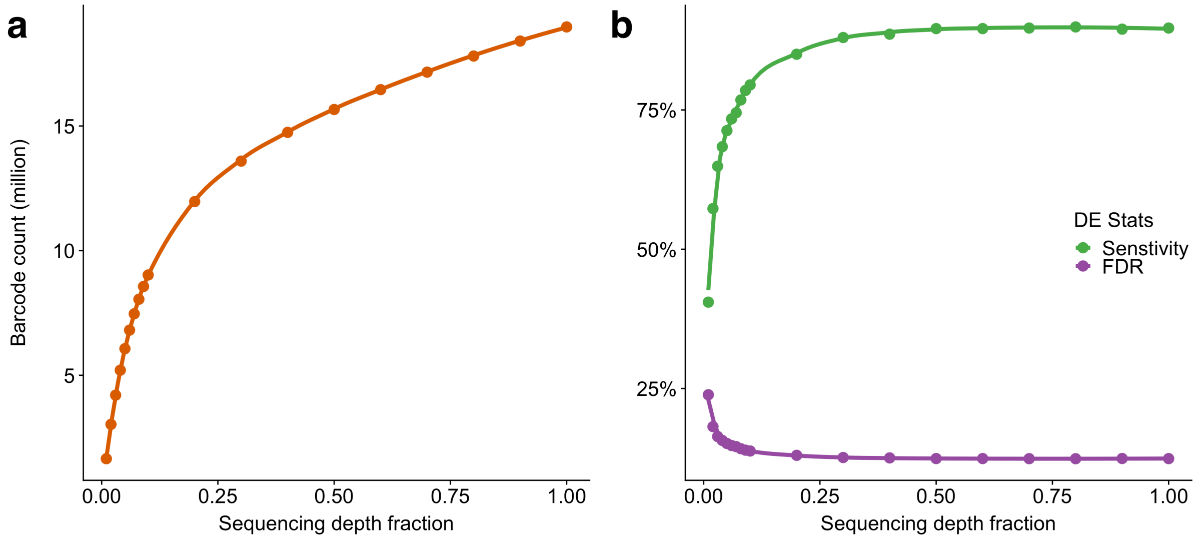

Fig S3: Downsampling of sequencing depth. From reads generated from the human/mouse control experiment, a fraction of reads was randomly selected and used in downstream analysis. The results showed that the sequencing depth used in this experiment was close to saturation. (a) Numbers of barcodes detected from a fraction of reads. (b) Differential expression analysis stats (sensitivity and false discovery rate) calculated from a fraction of reads.

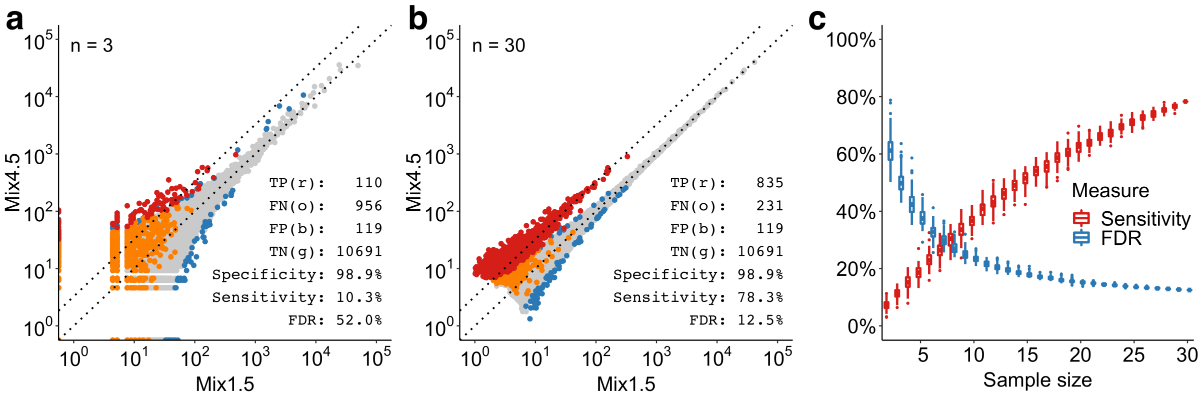

Fig S4: Performance evaluation of Decode-seq at 3-fold change level using human/mouse RNA mixes. (a) Differential expression analysis with 3 pairs of replicates. True positive (TP, red dots): mouse genes which were called DE; false negative (FN, orange dots): mouse genes which were called non-DE; true negative (TN, gray dots): human genes which were called non-DE; false positive (FP, blue dots): human genes which were called DE. Specificity = TN/(TN+FP), and it was fixed to 98.9% in all calculation. Sensitivity = TP/(TP+FN). False discovery rate = FP/(TP+FP). The sensitivity was only 10.3% and the false discovery rate was 52%. (b) DE analysis with 30 pairs of replicates. The sensitivity increased to 78.3% and the false discovery rate dropped to 12.5%. (c) DE performance related to replicate number calculated by random downsampling of 30-pair data. Each replicate number was calculated 100 times. Sensitivity and false discovery rate were improved dramatically when the number of replicates increased.

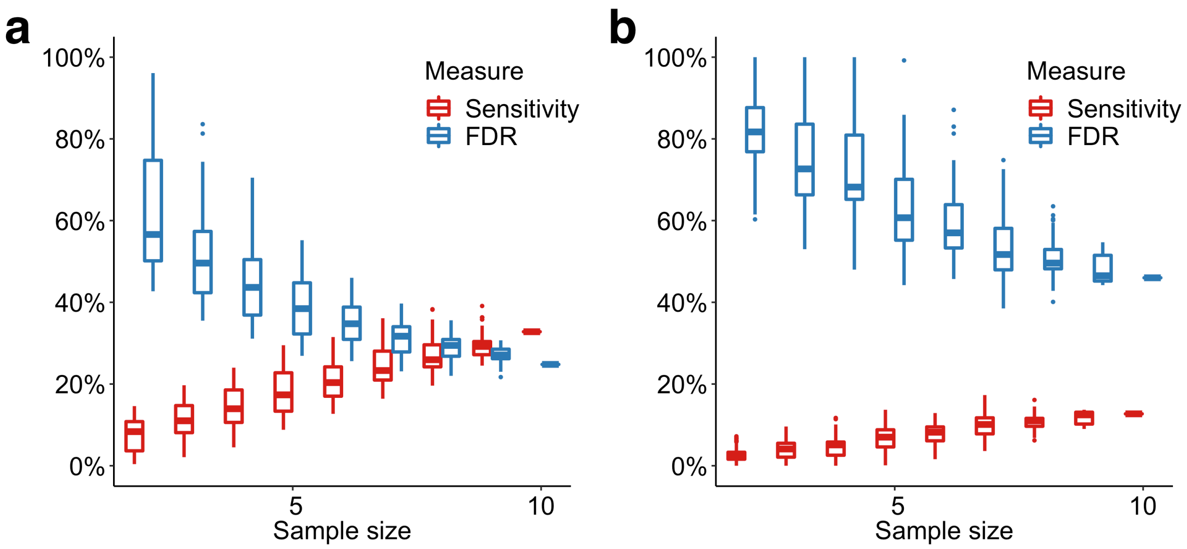

Fig S5: Performance evaluation of Decode-seq with 10 ng and 1 ng RNA at 5-fold change level. 10 pairs of replicates were used in these experiments. The performance was not as good as 100 ng, but the trend was the same, more replicates improved DE analysis. (a) Starting material: 10 ng. (b) Starting material: 1 ng.

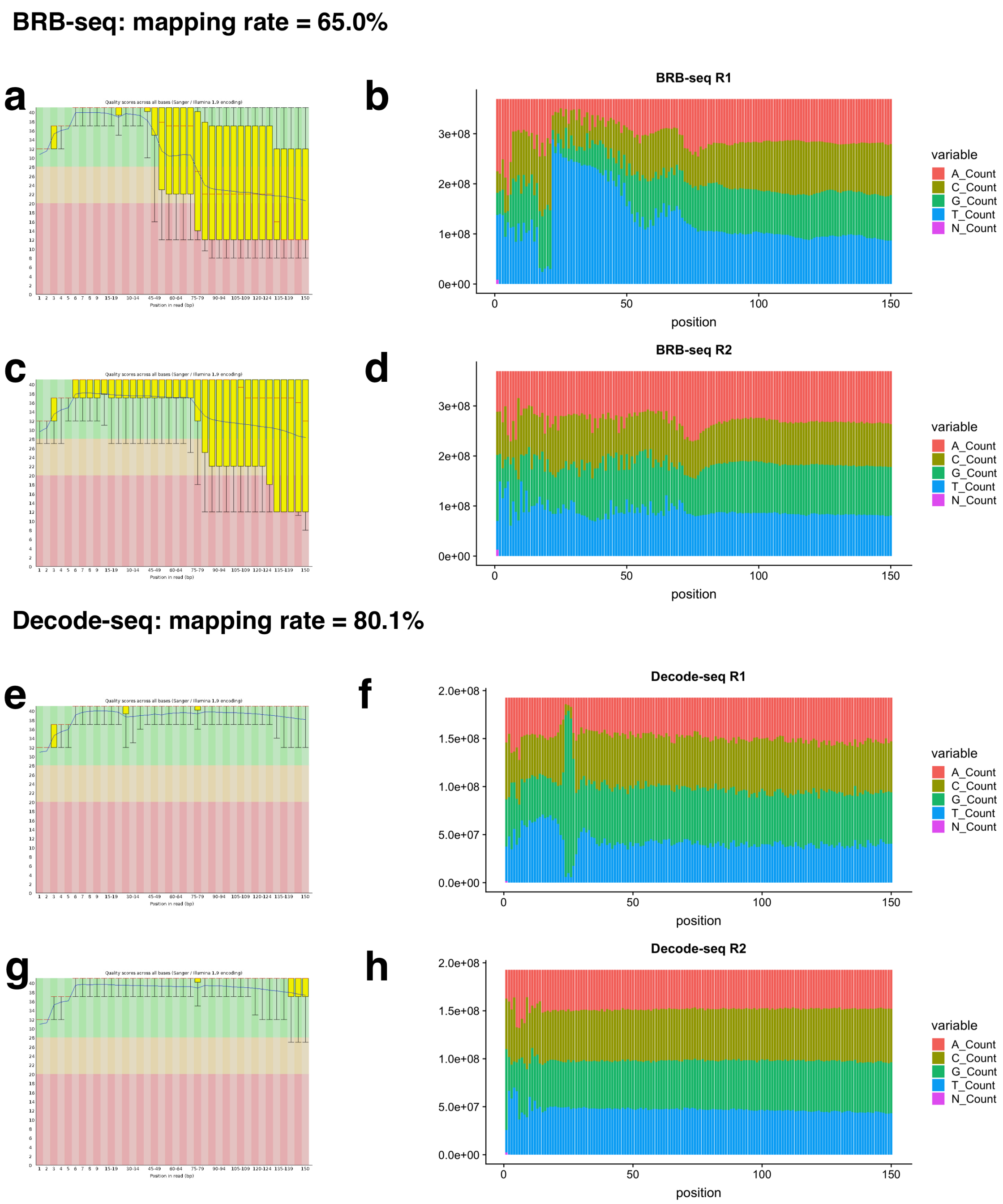

Fig S6: Sequencing quality scores and nucleotide distribution of BRB-seq and Decode-seq. a,c,e,g represent the per base sequence quality of read 1 and read 2 of BRB-seq and Decode-seq. b,d,f,h represent the nucleotide distribution of read 1 and read 2 of BRB-seq and Decode-seq. For BRB-seq, position 1–6 is the USI barcode, position 7–21 is the UMI barcode, and the rest is the cDNA sequence. For Decode-seq, position 1–6 is the USI barcode, position 7–23 is the UMI barcode, position 24–26 is the three Guanines, and the rest is the 5' end cDNA sequence.

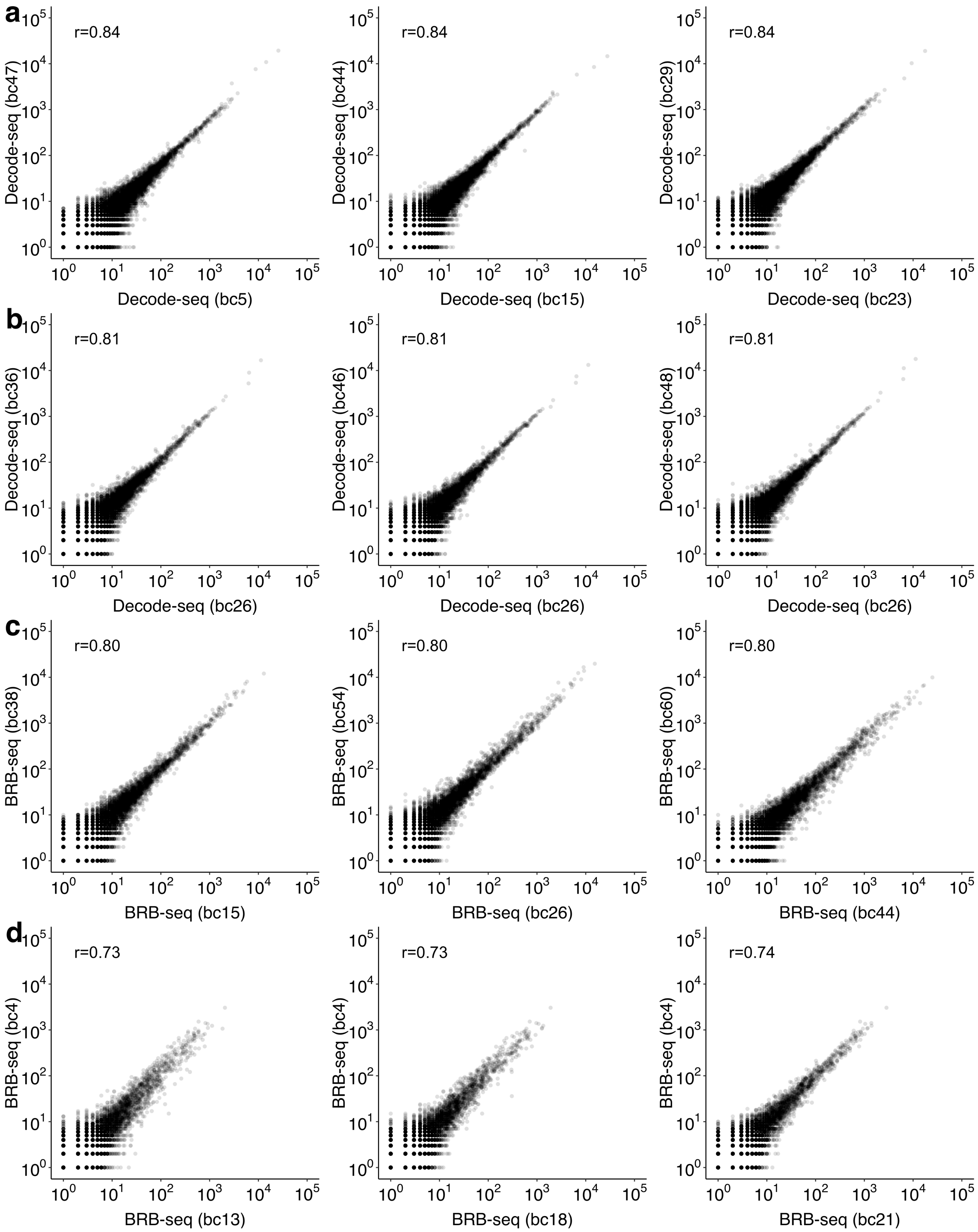

Fig S7: Spearman's correlations of human gene UMI counts between technical replicates of Decode-seq and BRB-seq. (a),(b) Correlation of Decode-seq replicates with three pairs of mean correlation and three of the worst correlation. (c),(d)Correlation of BRB-seq replicates with three pairs of mean correlation and three of the worst correlation.

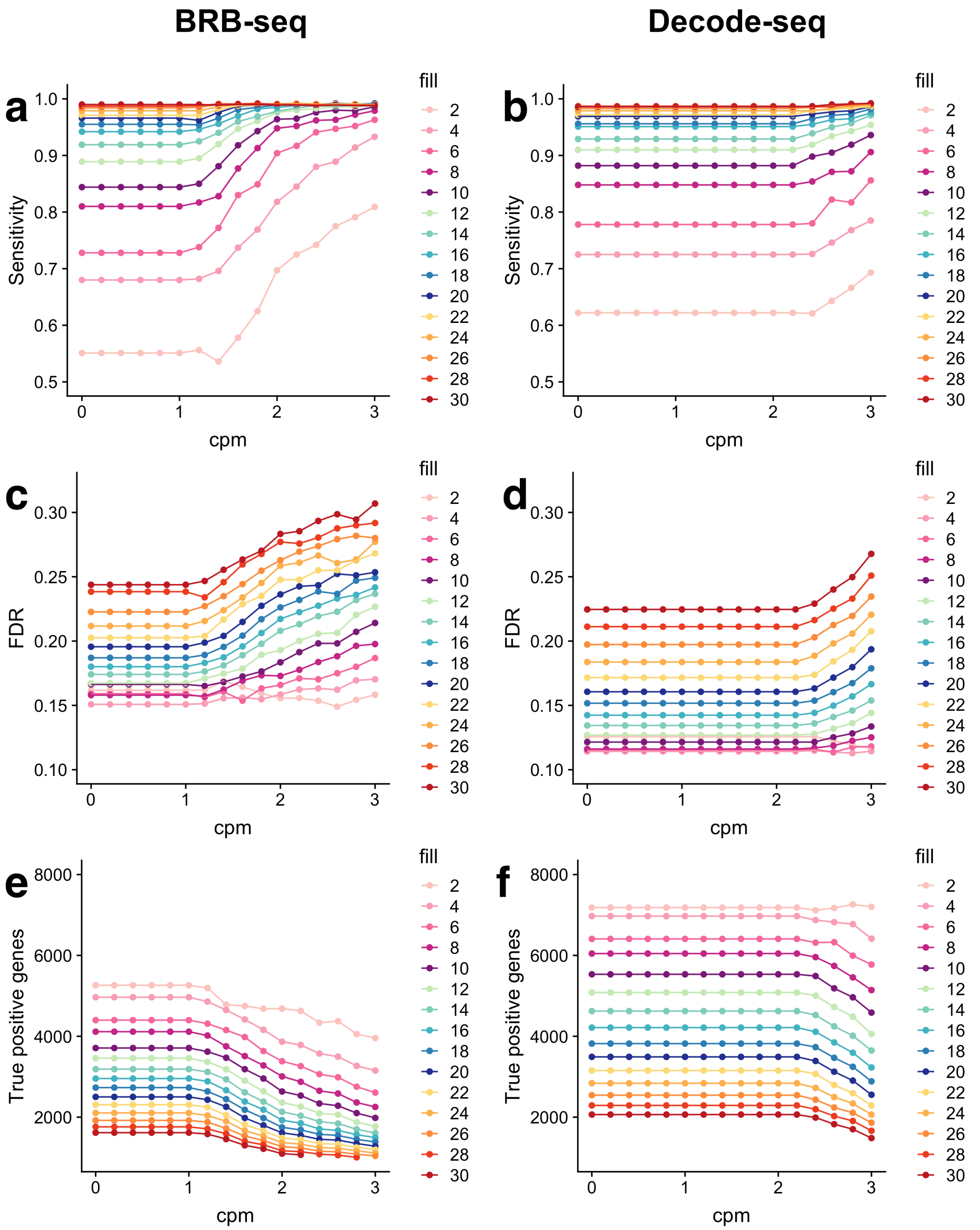

Fig S8: DE performance of BRB-seq and Decode-seq when using different edgeR filtering parameters. The two parameters (cpm and fill) means a gene will be retained if it is only expressed more than a CPM value(cpm) in more than a particular number (fill) of samples in each group. Using various combinations of cpm and fill, the sensitivity, FDR and true positive gene number is calculated.

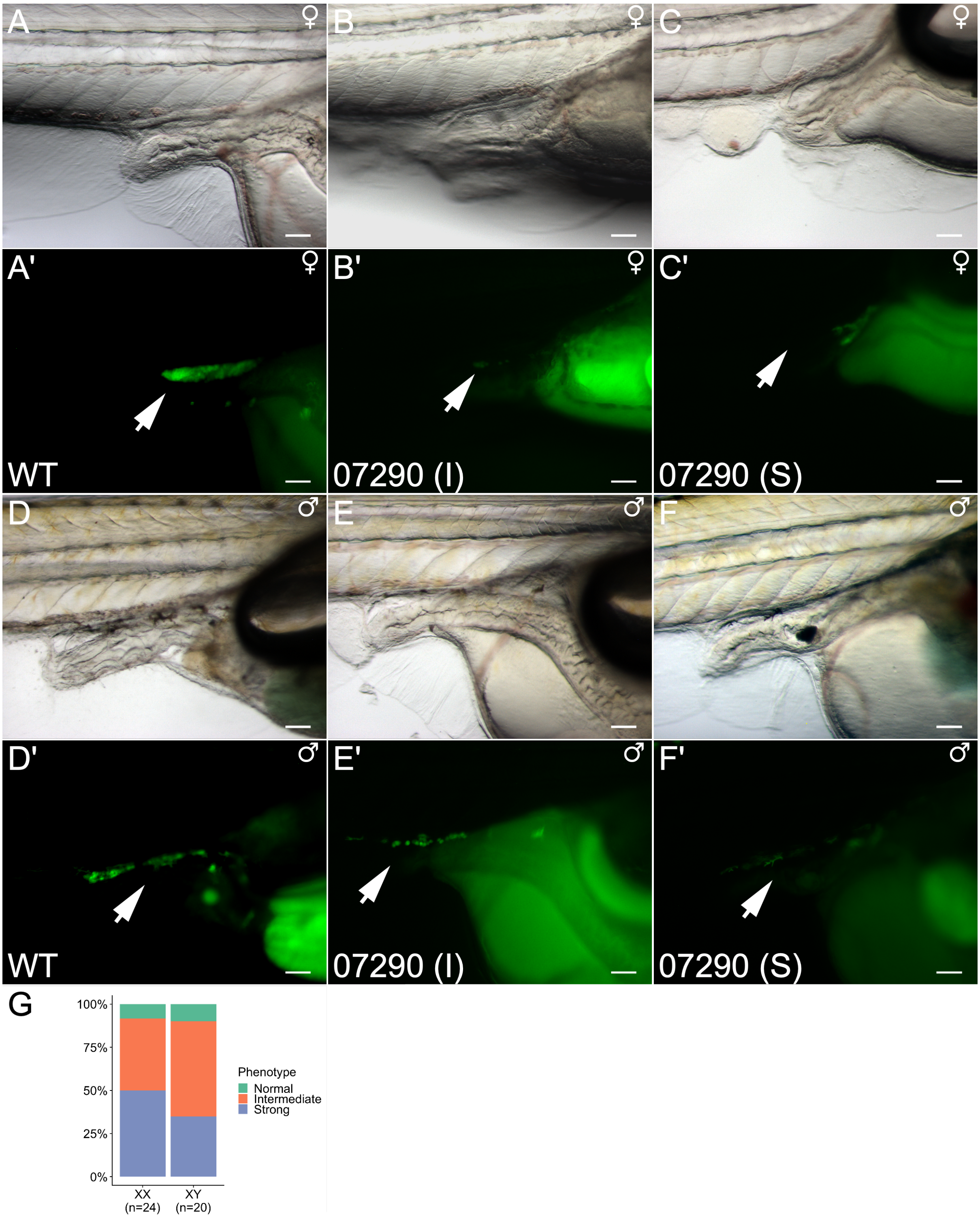

Fig S9: Genetic knockout of ENSORLG00000007290 by four-guide Cas9 RNP. Medaka fry at stage 39 (just hatching) were collected, imaged and scored. Arrows indicate GFP labeled germ cells. The phenotype of mutants was classified into three categories: normal, intermediate, and strong. Scale bar: 100 µm. (A-F) Bright-field images. (A'-F') Fluorescent images of the same specimen. (A-C) Female fry. (D-F) Male fry. (A/D) Wild type fry, clusters of germ cells with GFP signals are visible, and the female carries stronger signal than male. (B/E) Intermediate phenotype, in which germ cells are largely depleted. (C/F) Strong phenotype, in which germ cells are almost completely disappeared. (G) Percentage of three different phenotypes in male and female.
