# Supplementary material for: Decode-seq: a practical approach to improve differential gene expression analysis
Source: Genome Biol. 2020 Mar 23;21:66. doi: 10.1186/s13059-020-01966-9 (PMC7087377; doi:10.1186/s13059-020-01966-9)
Supplement: Supplementary file 3 — Additional file 3 Supplementary methods. [file 13059_2020_1966_MOESM3_ESM.pdf]

Decode-seq: a practical approach to improve differential gene expression analysis

Supplementary Methods

Yingshu Li[1,2,3], Hang Yang[1,2,3], Hujun Zhang[1,2,3], Yongjie Liu[1,2,3], Hanqiao Shang[1,2], Herong Zhao[1,2,3], Ting Zhang[1,2], and Qiang Tu[1,2,3,*]

[1]*State Key Laboratory for Molecular and Developmental Biology, Institute of Genetics and Developmental Biology, Innovation Academy for Seed Design, Chinese Academy of Sciences, Beijing 100101, China*
[2]*Key Laboratory of Genetic Network Biology, Institute of Genetics and Developmental Biology, Chinese Academy of Sciences, Beijing 100101, China.*
[3]*University of Chinese Academy of Sciences, Beijing 100049, China*

Decode-seq design

In Decode-seq, each cDNA molecule was barcoded with two short sequences, a unique sample identifier (USI) and a unique molecule identifier (UMI), using the template switching technology (Additional file 1: Fig. S2). The first step was reverse transcription and template switching. The 5bc-RT primer contains a common linker (yellow box in the Additional file 1: Fig. S2a) and poly-T sequence (green box), and its function is to initialize the reverse transcription and add the common linker at the cDNA end corresponding to the 3' of the transcript. The 5bc-TSO primer contains a common linker (yellow box), 6bp USI barcode (red box), 17bp UMI barcode (orange box), and GGG. Its function is to initialize template switching and add barcodes and the common linker at the cDNA end corresponding to the 5' of the transcript. The second step is PCR pre-amplification. It was initialized by the single-PCR primer which matched the common linker at both ends of the cDNA. The next is the tagmentation reaction. Tn5 transposase cleaved and tagged the double-stranded cDNA with a universal oligo (skyblue box). The final step is 5' enrichment PCR. Two primers (P5R1 and P7) matched the common linker and Tn5 universal oligo respectively, so that only sequences of 5' end of transcripts were amplified for high-throughput sequencing. These sequences carried three pieces of information: sample identification, reverse transcription product identification, and gene identification. They were also tagged with Illumina common sequencing primers. Therefore, the product of this step is sequencing-ready cDNA fragments. The single-PCR primer and P5R1 primer were used in SCRB-seq [1]. All primers used are listed in Additional file 2: Table S2.

Cell culture and collection

HEK293T cells were cultured in DMEM medium supplemented with 2 mM L-glutamine, 100 units/ml Penicillin-Streptomycin, and 10% (v/v) fetal bovine serum (FBS). Mouse MEF (C57BL/6 x DBA/2) cells were cultured in DMEM medium supplemented with 2 mM L-glutamine, 100 units/ml Penicillin-Streptomycin, and 15% (v/v) FBS. Cells were collected by pipetting into a 15 mL tube and spin down in a centrifuge.

Fish

Two medaka strains, d-rR-Tg(olvas-GFP) [2] and Qurt, were used in this study. Both strains were obtained from Japan NBRP Medaka. Two strains were crossed and selected, so that the offspring strain carries both sexually dimorphic pigmentation and germ cell specifically expressed GFP. Fish were maintained in fresh water at 28 °C under photo-periodically regulated conditions (14 h light and 10 h dark).

Tissue micro-dissection

Gonadal fragments, which include gonads, intestines, and body trunks, were micro-dissected from medaka fry of stage 39 (just hatching) with knives and forceps. Each single gonadal fragments was put in TRIzol (Life Technologies, cat. 15596-018) and snap froze in liquid nitrogen then stored at −80 °C for later use. The rest piece of fish was used for PCR genotyping to confirm the gender. The primers were listed in Additional file 2: Table S2.

RNA isolation

RNA was extracted from medaka tissues using Both TRIzol and RNeasy Micro Kit (Qiagen, cat. 74004) with a modified protocol. Tissue samples were first homogenized in TRIzol, then repeated the freeze-thaw cycle 3 times with shaking in the interim to ensure complete lysis of the sample. Next, the phase separation step was performed according to the standard TRIzol protocol. The extracted RNA samples were transferred to the RNeasy Micro columns and processed following the RNeasy Micro protocol. In this way, complete lysis and high RNA yield from small tissue samples were ensured.

For cells, 1 ml TRIzol reagent was added directly to frozen cell samples and mix by pipetting until the cell pellet thawed. Following steps were performed according to the standard TRIzol protocol.

Decode-seq library construction

A Decode-seq library was built in four steps: (1) reverse transcription and template switching, (2) PCR pre-amplification, (3) Tagmentation, and (4) 5' enrichment PCR amplification.

The RNA mix was prepared with 1.3 µl RNA sample, 1 µl ERCC RNA Spike-In Control Mixes (Life Technologies, cat. 4456740, 1 µl 1/1000 dilution for 100 ng RNA sample), 1 µl of 10 µM 5bc-RT primer and 1 µl of 10 mM dNTP mix (Thermo Fisher, cat. R0192). The RNA mix was incubated for 3 min at 72 °C and then kept on ice until the next step.

The reverse transcription reaction was adapted from the Smart-seq2 protocol [3]. The RT mix was prepared with 2 µl of 5× Superscript II first-strand buffer, 2 µl of 5 M Betaine to a final concentration of 1 M, 0.5 µl 100 mM DTT to a final concentration of 5 mM, 0.06 µl 1M $MgCl_2$ to a final concentration of 6 mM, 0.5 µl SuperScript II reverse transcriptase (200 U/µl), 0.25 µl of 40 U/µl Recombinant RNase Inhibitor (TAKARA, cat. 2313A) and 0.29 µl Nuclease-free water (Ambion, cat. AM9932). 5.6 µl RT mix and 0.1 µl of 100 µM TSO primer were added to each RNA mix and the sample was incubated for 90 min at 42 °C, 10 cycles of 2 min at 50 °C, 2 min at 42 °C, 15 min at 70 °C. The product was kept at 4 °C until the next step.

For the PCR pre-amplification, 10 µl of the first-strand reaction was mixed with 12.5 µl of 2× KAPA HiFi HotStart Ready Mix (KAPA Biosystems, cat. KK2601), 0.25 µl of 10 µM single-PCR primer (final concentration 0.1 µM), and 2.25 µl Nuclease-free water, to a final volume of 25 µl. Samples were incubated 3 min at 98 °C followed by 10 cycles at 98 °C for 20 s, 64 °C for 15 s, 72 °C for 6 min, then a final extension at 72 °C for 5 min. The cDNA product was purified with Quick PCR Purification Kit (GeneOn Biotechnology, cat. GO-PCRF-100).

For the tagmentation reaction, the cDNA was processed using the Nextera XT DNA Library Prep Kit (Illumina, cat. FC-131-1024) according to the manufacturer's protocol, followed by the 5' enrichment PCR amplification. In this step, the index 2 primer P5 was replaced with customized primer P5R1. The product was the sequencing-ready cDNA library.

Lastly, the library was size selected and purified by a three-step procedure. Contaminants such as primers, dimers, salts, and small amplicons were removed by a quick PCR purification beads kit (GeneOn Biotechnology, cat. GO-PCRF-100). Then the library was roughly selected for about 300 - 450 bp by a gel extraction beads kit (GeneOn Biotechnology, cat. GO-GELU-100). The final step is the size selection with 0.7× ratio of SPRIselect beads (Beckman Coulter, cat. B23317). Quality and yield of the library were determined with an Agilent 2100 Bioanalyzer.

Decode-seq libraries are compatible with common Illumina sequencing systems with universal primers. In this study, all libraries were sequenced by commercial service provided by Annoroad Gene Technology Corporation (Beijing, China).

Data analysis

FastQC (v0.11.3) was used for quality control checks of sequencing reads. FASTX-Toolkit (v0.0.13) was used to calculate the nucleotide distribution on every single base pair position (Additional file 1: Fig. S6). In read 1, position 1–6 is the USI barcode, position 7–23 is the UMI barcode, followed by the three Guanines and 5' end cDNA sequence. Four types of nucleotides in UMI barcode and cDNA sequence were uniformly distributed. The nucleotide distribution in USI was almost evenly because multiple samples were pooled in one library, and the USI barcodes were designed for a balanced nucleotide distribution.

A bioinformatics pipeline was developed to analyze Decode-seq data (Supplementary Data). The processing includes: sequencing reads processing and filtering, genome mapping, quantification based on barcode counts. First, R1 reads must match one of the USIs used in the experiment at position 1–6, and has three Guanines at position 24–26. Any reads which did not meet the pattern were filtered. USI and UMI sequences were extracted for later quantification step. Then, R2 reads were mapped to the reference genomes (ENSEMBL human GRCh38, mouse GRCm38, and medaka ASM223467v1) with STAR (v2.5.2a) [4] using default parameters. Put all these pieces of information, R2 decided the origin gene; USI from R1 decided the original sample identity; UMI from R1 decided the original transcript identity. For each given gene, the number of UMI species, not the number of reads mapped, was used for quantification. Finally, the pipeline output a matrix table of quantification of the given gene in the given sample. Reads and mapping statistics were listed in Additional file 5: Table S3.

DE analysis was performed with the edgeR package (v3.8) using the generalized linear model method.

In the human-mouse RNA experiment, appropriate filtering conditions were set to remove genes with very low expression level (count per million >1 at least in 16 replicates in each group). Mouse/human reads percentage in two different mixes (mix5, mix1) were calculated, and they were very close to the designed percentages. Correlations between every sample pair were also calculated (Spearman's correlation) (Fig. 1d). The median correlation coefficient is 0.96. Since by experimental design, the real positive (mouse gene) and real negative (human gene) were known, we can calculate true positive (TP), true negative (TN), false positive (FP), and false negative (FN) rates, then other statistics including sensitivity, specificity, and false discovery rate etc can be calculated. For sample size downsampling analysis, 2–29 pairs of replicates were randomly selected for the DE analysis, and each replicate number were repeated for 100 times. For sequencing depth downsampling analysis, 1–100% sequencing reads were randomly selected for the DE analysis. In the medaka experiment, top 300 genes ranked by adjusted p values were selected for further analysis.

DEG Validation

First, qPCR was performed to validate the sexually dimorphic expression of identified DEGs. Female and male fry (stage 39) were collected and homogenized immediately in TRIzol for RNA extraction as discussed above. cDNA synthesis was carried out using iScript cDNA Synthesis Kit (Bio-Rad, cat. 170-8890). qPCR was performed on the total cDNA output with 4 repeats using Power SYBR Green PCR Master Mix (Applied Biosystems, cat. 4367659) and processed on an Applied Biosystems QuantStudio 12K Flex real-time PCR systems (Life Technologies), allowing amplification of 384 samples per run. All primer sequences were listed in Additional file 2: Table S2. Statistical analysis for comparison of two groups was performed using two-tailed unpaired Student's t-test.

In situ hybridization chain reaction (HCR) was performed following the published protocol [5]. Probes targeting three genes were used in the experiment: *vasa* as the germ cell marker, *sox9b* as the somatic cell marker, and *cd74a*, the new identified DEG. All HCR reagents were ordered from Molecular Instruments (https://www.molecularinstruments.com). Images of medaka fry were collected with a spectral confocal and multiphoton microscope (Carl Zeiss, LSM 780 NLO).

CRISPR/Cas9 knockout

The function of a novel DEG was validated using a rapid knockout method to generate F0 mutants [6, 7]. The gRNA target sites were designed using CRISPrscan (http://www.crisprscan.org/). Four gRNA targets were used in this study (Additional file 2: Table S2). The gRNA templates were amplified by PCR, purified by Universal DNA Purification Kit (Tiangen Biotech, cat. DP214-03) and *in vitro* transcribed by MAXIscript T7 *in vitro* Transcription Kit (Thermo Fisher, cat. AM1314). The gRNAs were purified using RNA Clean Kit (Tiangen Biotech, cat. DP412). The Cas9 Nuclease protein (New England Biolabs, cat. M0386T) solution (20 µM) containing glycerol was stored at −20 °C. The Cas9 protein was mixed with gRNA to generate Cas9 RNP complex. 0.05% phenol red dye was added for visualization of microinjection. The Cas9 RNP complex was incubated at 37 °C for 5 minutes before injection. Microinjection was performed by injecting the complex into medaka embryos at the one-cell stage using MPPI-3 injector (ASI USA). The Cas9 Nuclease (500 ng/µl) and gRNA mRNA (400 ng/µl) were simultaneously injected into the embryos. After injection, embryos were maintained in embryo culture medium at 28 °C until stage 39 reached for collection and scoring. Images were collected with a fluorescent stereomicroscope (Leica M205A).

Comparison with BRB-seq

The BRB-seq library was built according to the published BRB-seq protocol using the same RNA mix samples and input amount as Decode-seq (Mix1 and Mix5, 100ng). Illumina Nextera XT DNA Library Prep Kit was used for the tagmentation steps. All primers can be found in the original BRB-seq paper, and from the original 96 sample barcodes, 60 barcodes were selected for 60 replicates (Additional file 2: Table S2).

For an unbiased comparison between BRB-seq and Decode-seq, we generated almost equal amount of sequencing data for the two libraries BRB-seq 369M and Decode-seq 356M reads). Count-based quantification was done using the pipline we developed, which is compatible with both BRB-seq and

Decode-seq. For the DE analysis, a variety of data filtering parameters for edgeR was tested and the results were shown in the Additional file 1: Fig. S7.

References

[1] Soumillon M, Cacchiarelli D, Semrau S, van Oudenaarden A, Mikkelsen TS. Characterization of Directed Differentiation by High-Throughput Single-Cell RNA-Seq. bioRxiv. 2014 Mar;p. 003236.

[2] Tanaka M, Kinoshita M, Kobayashi D, Nagahama Y. Establishment of Medaka (Oryzias Latipes) Transgenic Lines with the Expression of Green Fluorescent Protein Fluorescence Exclusively in Germ Cells: A Useful Model to Monitor Germ Cells in a Live Vertebrate. Proc Natl Acad Sci USA. 2001 Feb;98(5):2544–2549.

[3] Picelli S, Björklund ÅK, Faridani OR, Sagasser S, Winberg G, Sandberg R. Smart-Seq2 for Sensitive Full-Length Transcriptome Profiling in Single Cells. Nat Methods. 2013 Nov;10(11):1096–1098.

[4] Dobin A, Davis CA, Schlesinger F, Drenkow J, Zaleski C, Jha S, et al. STAR: Ultrafast Universal RNA-Seq Aligner. Bioinformatics. 2013 Jan;29(1):15–21.

[5] Choi HMT, Calvert CR, Husain N, Huss D, Barsi JC, Deverman BE, et al. Mapping a Multiplexed Zoo of mRNA Expression. Development. 2016 Jan;143(19):3632–3637.

[6] Sawamura R, Osafune N, Murakami T, Furukawa F, Kitano T. Generation of Biallelic F0 Mutants in Medaka Using the CRISPR/Cas9 System. Genes Cells. 2017 Aug;22(8):756–763.

[7] Wu RS, Lam II, Clay H, Duong DN, Deo RC, Coughlin SR. A Rapid Method for Directed Gene Knockout for Screening in G0 Zebrafish. Dev Cell. 2018 Jul;46(1):112–125.e4.
