# Supplementary material for: Decode-seq: a practical approach to improve differential gene expression analysis
Source: Genome Biol. 2020 Mar 23;21:66. doi: 10.1186/s13059-020-01966-9 (PMC7087377; doi:10.1186/s13059-020-01966-9)
Supplement: Supplementary file 4 — Additional file 4 Supplementary data: the experimental protocol and computational analysis pipeline for decode-seq. [file 13059_2020_1966_MOESM4_ESM.zip › Decode-seq-master/decode-seq_protocol.pdf]

Decode-seq Protocol

Tu Lab, IGDB, CAS (http://tulab.genetics.ac.cn)

v1 (201908)

Materials

Reagents

1. TRIzol (Life Technologies, cat. 15596-018)
2. RNeasy Micro Kit (Qiagen, cat. 74004)
3. ERCC RNA Spike-In Control Mixes (Life Technologies, cat. 4456740)
4. dNTP mix (Thermo Fisher, cat. R0192)
5. Recombinant RNase Inhibitor (TAKARA, cat. 2313A)
6. Nuclease-free water (Ambion, cat. AM9932)
7. KAPA HiFi HotStart Ready Mix (KAPA Biosystems, cat. KK2601)
8. Quick PCR Purification Kit (GeneOn Biotechnology, cat. GO-PCRF-100)
9. Nextera XT DNA Library Prep Kit (Illumina, cat. FC-131-1024)
10. Quick PCR purification beads kit (GeneOn Biotechnology, cat. GO-PCRF-100)
11. Gel extraction beads kit (GeneOn Biotechnology, cat. GO-GELU-100)
12. SPRIselect beads (Beckman Coulter, cat. B23317)
13. Superscript II reverse transcriptase (Invitrogen, cat. 18064-014)
14. Qubit dsDNA high-sensitivity (HS) kit (Invitrogen, cat. Q32851)

Reagent setup

1. 5bc-TSO primer: dissolve in TE buffer, to a final concentration of 100 µM. Store at −80 °C for 6 months. Avoid repeated freeze-thaw cycles.
2. 5bc-RT primer and single-PCR primer: dissolve in TE buffer, to a final concentration of 100 µM. Store at −20 °C for 6 months. Dilute these two primers to 10 µM by nuclease-free water and mix well before use.

Procedure

RNA preparation

Any RNA extraction protocol with sufficient RNA yield is feasible. For small tissue samples, we recommend using both TRIzol and RNeasy Micro Kit with a modified protocol, by which complete lysis and high RNA yield from small tissue samples are ensured. The steps are shown below:

1. Tissue samples are first homogenized in TRIzol, then freeze–thawed 3 times with shaking in the interim to ensure complete lysis.
2. The phase separation step is performed according to the standard TRIzol protocol.
3. The extracted RNA samples are transferred to the RNeasy Micro columns and processed following the RNeasy Micro protocol.
4. Add ERCC if required:

RNA per action	ERCC dilution	ERCC vol
100 ng	1/1000	1 µl
10 ng	1/10000	1 µl
1 ng	1/100000	1 µl

Reverse Transcription

1. Prepare RNA mix: 2.3 µl RNA (containing ERCC if required), 1 µl of 10 µM 5bc-RT primer, 1 µl of 10 mM dNTP mix.
2. Vortex mix, spin down (700 g, 10 s, RT) and immediately put on ice.
3. 72°C for 3 min, immediately put on ice.
4. Spin down (700 g, 10 s, RT), immediately put on ice (oligo-dT hybridized to polyA).
5. Prepare RT mix (5.6 µl/action).

Reagent	Volume (µl)	Final concentration
Superscript II first-strand buffer (5×)	2.00	1×
Betaine (5 M)	2.00	1 M
DTT (100 mM)	0.50	5 mM
MgCl2 (1 M)	0.06	6 mM
Nuclease-free water	0.29	–
SuperScript II reverse transcriptase (200 U/µl)	0.50	100U
RNase inhibitor (40 U/µl)	0.25	10U
Total Volume	5.60	

6. Add 0.1 µl TSO (100 µM) into the sample.
7. Add 5.6 µl RT mix into the sample (total 10 µl), gently pipet up and down a few times (usually 10 times) without forming bubbles.
8. Spin down (700 g, 10 s, RT), incubate in a thermal cycler with a heated lid, as below (2.5 hours).

cycle	temperature (°c)	time	purpose
1	42	90 min	RT and template-switching
2–11	50	2 min	Unfolding of RNA secondary structures
	42	2 min	Completion/continuation of RT and template-switching
12	70	15 min	Enzyme inactivation
13	4	Hold	Safe storage

Pre-amplification PCR

1. Prepare the PCR mix

Reagent	Volume (µl)	Final concentration
KAPA HiFi HotStart ReadyMix (2×)	12.50	1×
Single-P primer (10 µM)	0.25	0.1 µM
Nuclease-free water	2.25	–
Total Volume	25	–

2. Add 15 µl PCR mix to the first-strand reaction (10 µl), vortex, spin down (700 g, 10 s, RT).
3. PCR, as below (total 25 µl, 2.5 hours).

- Note: 18 cycles for single eukaryotic cells can obtain ~1–30 ng of amplified cDNA. Here we use 10 cycles for 100ng input RNA sample.
- PCR products can be stored at −20 or −80 °C for 6 months or longer.

cycle	Denature	anneal	extend	Hold
1	98 °C, 3 min	-	-	-
2–11	98 °C, 20 s	67 °C, 15 s	72 °C, 6 min	–
12	–	–	72 °C, 5 min	–
13	–	–	–	4 °C

cDNA purification

1. Take 40 µl amplified cDNA, and purify with Quick PCR Purification Kit.
2. Quantification: Qubit 3.0, dilute to 0.2 ng/µl.

Library construction (Nextera XT)

Tagmentation (25 µl)

1. Thaw buffer ATM, TD and DNA samples on ice, ensure NT (RT) no precipitates.
2. Add 10 µl TD, 5 µl DNA sample (total 1 ng), pipette to mix.
3. Add 5 µl ATM, pipette to mix.
4. 280 g at 20°C for 1 minute.
5. Run the tagmentation program as below.

Choose the preheat lid option	
55°C	5min
10°C	Hold

6. Add 5 µl NT, pipette to mix.
7. 280 g at 20°C for 1 minute.
8. RT for 5 minutes.

Amplification

1. Thaw indexes at RT for 20 minutes. Invert tube to mix and centrifuge briefly. Thaw NPM/KAPA on ice.
2. For general experiments, use the KAPA method as below:

Reagent	Volume (µl)	Final concentration
DNA	25 µl	
Index 1 i7 primer (27 µM)	1 µl	0.5 µM
P5Read1 (instead of Index 2 i5) primer (27 µM)	1 µl	0.5 µM
KAPA	27 µl	1 ×
	pipette to mix	
Total Volume	54 µl	

1. (Optional) NPM method as below

Reagent	Volume (µl)	Final concentration
DNA	25 µl	
Index 1 i7 primer (5 µM)	5 µl	0.5 µM
P5Read1 (instead of Index 2 i5) primer (5 µM)	5 µl	0.5 µM
NPM (Nextera PCR master mix)	15 µl	
	pipette to mix	
Total Volume	50 µl	

4. 280 g at 20°C for 1 minute.
5. Run the PCR program as below.

cycle	Denature	anneal	extend	Hold
1	–	–	72°C, 3min	–
2	95 °C, 30 s	–	–	–
3-18	95 °C, 10 s	55 °C, 30 s	72 °C, 30 s	–
19	–	–	72 °C, 5 min	–

cycle	Denature	anneal	extend	Hold
21	–	–	–	10 °C

Clean up library

The library is size selected and purified by a three-step procedure:

1. Library purification by the quick PCR purification beads kit.
2. Rough library selection for about 300 - 450 bp by the gel extraction beads kit.
3. Size selection with 0.7× ratio of SPRIselect beads.
4. Quality and yield of the library are determined with an Agilent 2100 Bioanalyzer.
