# Supplementary material for: Decode-seq: a practical approach to improve differential gene expression analysis
Source: Genome Biol. 2020 Mar 23;21:66. doi: 10.1186/s13059-020-01966-9 (PMC7087377; doi:10.1186/s13059-020-01966-9)
Supplement: Supplementary file 5 — Additional file 5 Review history. [file 13059_2020_1966_MOESM5_ESM.docx]

**Review History**

**First round of review**

**Reviewer 1:**

This is a curious paper. The paper is presented as being about replicate numbers and RNA-seq analysis, but the paper doesn't actually have anything novel to say about those things. It is already obvious and well known that having more biological replicates will increase statistical power to detect differentially expressed genes, so there is nothing novel in demonstrating that.

The novelty of the paper is actually that the authors propose a UMI 5' orientated protocol for RNA-seq, which they call Decode-seq. I would have expected the paper to compare Decode-seq with regular RNA-seq, but no comparisons are made.

The paper claims the main advantage of Decode-seq to be the ability to multiplex samples on to a single lane of a sequencer, but multiplexing is already a routine procedure for regular RNA-seq processing. My institution performs RNA-seq runs every day using NextSeq and HiSeq machines and regular Illumina protocols. We use multiplexing for almost every run and have done so since the early days of RNA-seq. We can multiplex up to 48 RNA samples into a single sequencing lane. The authors of the current paper seem to be unaware that multiplexing is already available.

The paper claims that Decode-seq can handle small starting quantities of RNA, but no evidence is provided that this is so. Low input protocols such as Smart-seq are already routinely available for regular RNA-seq.

The paper claims that 5' enrichment is an advantage, but it appears to me to be a disadvantage because it prevents the RNA-seq data from being used to detect splicing events or transcripts.

I had a quick look at the count files deposited to GEO as series GSE130014. At first glance, these files do not look to be very high quality. The only genes are with high counts are either spike-ins or ribosomal RNA, neither of which would be of interest in a real analysis. The regular genes all appear to have very low read counts.

**Reviewer 2:**

The authors present a method for using molecular barcoding to help reduce costs of bulk RNA-seq and thereby allow higher number of biological replicates. The human/mouse mixture experiment indicates the advantage of adding many replicates, in increasing the precision of measurements.

Major comments:

My biggest concern is that the manuscript does not adequately compare itself to other existing approaches, or describe differences between itself and existing approaches. Since January 2018, a preprint on bioRxiv (doi: 10.1101/256594) has described "Bulk RNA Barcoding and sequencing (BRB-seq)", which appears similar in design and application to Decode-seq, and this was recently published in Genome Biology (10.1186/s13059-019-1671-x). The final publication of BRB-seq has extensive description of the various approaches for molecular barcoding for single and bulk RNA-seq, including citing seven publications where SCRB-seq including multiplexing has been applied to bulk RNA samples (references 14-20 in the BRB-seq publication). A major difference is that BRB-seq produces 3' tagged libraries whereas Decode-seq produces 5' tagged libraries. Decode-seq therefore may be a valuable contribution to the literature, but further comparison with existing approaches should be made, and a more complete description of the method with respect to other existing approaches at multiplexing bulk RNA-seq should be provided.

Some statements in the manuscript require much stronger evidence than presented. For example "We downsampled the sequencing depth and found 2.8M reads per sample was enough in this experiment". Sequencing is far too complicated for such a statement to be made. Lowly expressed differentially expressed transcripts will be invisible at such a depth, and so there is always a trade-off between higher replicates benefitting the precision of estimation for highly expressed transcripts, and higher sequencing depth benefitting the ability to observe lowly expressed transcripts.

Minor comments:

Why was the specificity fixed to 98.9%? This number seems arbitrary. The caption says that the specificity was improved, while also claiming it was fixed.

Why were the top 300 genes selected, instead of choosing a q-value or adjusted p-value threshold? How were these genes selected? How were the 25 genes chosen for validation selected?

**Authors Response**

**Point-by-point responses to the reviewers’ comments:**

**Reviewer #1:**

We are sorry that we did not make it clear in our first version about early/late multiplexing, UMIs, and relative concepts. In the current version, we added several paragraphs to clarify these in details. We appreciate the reviewer’s questions which helped us to improve the clarity of the manuscript.

*I would have expected the paper to compare Decode-seq with regular RNA-seq, but no comparisons are made.*

Decode-seq is a multiplexing method working with low-input RNA. For example, in the current study, the maximum amount of input RNA we tested is 100 ng. Regular RNA-seq requires RNA amount larger than this, often at μg level. The recent published BRB-seq is also a multiplexing method working with low-input RNA. Therefore, we think the comparison with BRB-seq is more relevant and informative, so we experimentally compared Decode-seq with BRB-seq thoroughly (see discussion above), and add the new results in the manuscript.

*The paper claims the main advantage of Decode-seq to be the ability to multiplex samples on to a single lane of a sequencer, but multiplexing is already a routine procedure for regular RNA-seq processing. My institution performs RNA- seq runs every day using NextSeq and HiSeq machines and regular Illumina protocols. We use multiplexing for almost every run and have done so since the early days of RNA-seq.*

*We can multiplex up to 48 RNA samples into a single sequencing lane. The authors of the current paper seem to be unaware that multiplexing is already available.*

We are sorry for not clarifying the difference between early and late multiplexing in the first version.

The commonly used multiplexing in the sequencing platform is late multiplexing. Samples are processed individually and tagged at a late step (e.g., PCR amplification, or adapter ligation). Some full-length scRNA-seq methods (e.g., Smart-seq) also use this strategy. Late multiplexing cannot reduce the time and cost of library preparation because each library is processed individually.

The main point of Decode-seq, and BRB-seq, is the early multiplexing. RNA samples are first reverse transcribed individually and tagged at this step, and then all samples are mixed and handled together for the downstream process. In this way, Decode-seq profiles dozens of RNA samples simultaneously, and reduced the cost of library construction to a small fraction of regular RNA-seq.

Most RNA-seq methods are compatible with the late multiplexing strategy, including Decode-seq. As long as libraries use different Illumina indexes, a large number of libraries can be sequenced in one lane. That is why we are able to sequence only 5-10Gb of one Decode-seq library for the QC purpose, as a large number of libraries (Decode-seq and other standard Illumina libraries) are sequenced together in one lane.

We added two paragraphs to clarify the difference between early and late multiplexing.

*The paper claims that Decode-seq can handle small starting quantities of RNA, but no evidence is provided that this is so. Low input protocols such as Smart-seq are already routinely available for regular RNA-seq.*

We had actually shown the experiments of 10 ng and 1 ng in Supplementary Figure 5. As discussed above, Smart-seq is a late multiplexing method which cannot reduce the cost of library construction.

*The paper claims that 5' enrichment is an advantage, but it appears to me to be a disadvantage because it prevents the RNA-seq data from being used to detect splicing events or transcripts.*

Again, this is a confusion of early/late multiplexing. Early multiplexing methods are able to tag one end of the transcript only; thus they sacrifice the information of full-length transcripts while they enormously minimize the library construction labor and cost and increases the multiplexity to the level of dozens, thousands or even more.

Late multiplexing methods pursue the other side of the information. To our knowledge, currently there is no method that could observe the full-length transcripts of dozens of samples simultaneously in one library. Researchers have to choose proper methods depending on the purpose of the experiment.

*I had a quick look at the count files deposited to GEO as series GSE130014. At first glance, these files do not look to be very high quality. The only genes are with high counts are either spike-ins or ribosomal RNA, neither of which would be of interest in a real analysis. The regular genes all appear to have very low read counts.*

Decode-seq is a UMI based method, which means the count table contains the number of distinct UMIs, not the number of reads. Many reads could be mapped to one gene, but they will be count as one if the corresponding UMIs are the same. That is why the numbers in UMI count tables often look very low.

In summary, we added several paragraphs in the main text to explain more details about early/late multiplexing and UMIs, and compared Decode-seq with BRB-seq.

We hope these revisions and responses could address all issues raised by Reviewer #1.

**Reviewer #2:**

We appreciate Reviewer #2’s critical and invaluable comments very much. According to these suggestions, we completely redid the experiments of figure 1, using both Decode-seq and BRB-seq with the same samples. We add more description of molecular barcoding, UMIs, the advantages/disadvantages compared with other methods. Besides, we fully agreed that there were several mistakes or misleading points in the previous version, and we have revised accordingly.

*My biggest concern is that the manuscript does not adequately compare itself to other existing approaches, or describe differences between itself and existing approaches. Since January 2018, a preprint on bioRxiv (doi: 10.1101/256594) has described "Bulk RNA Barcoding and sequencing (BRB-seq)", which appears similar in design and application to Decode- seq, and this was recently published in Genome Biology (10.1186/s13059-019-1671-x). The final publication of BRB-seq has extensive description of the various approaches for molecular barcoding for single and bulk RNA-seq, including citing seven publications where SCRB-seq including multiplexing has been applied to bulk RNA samples (references 14-20 in the BRB-seq publication).*

*A major difference is that BRB-seq produces 3' tagged libraries whereas Decode-seq produces 5' tagged libraries. Decode-seq therefore may be a valuable contribution to the literature, but further comparison with existing approaches should be made, anda more complete description of the method with respect to other existing approaches at multiplexing bulk RNA-seq should be provided.*

According to these comments, we set up the BRB-seq method in our lab, communicated with the authors for technical details, made human/mouse RNA mixes again, profiled these mixes again using both methods, and carefully compared the performance of Decode-seq and BRB-seq. The results and descriptions have been discussed in the main text and the letter above.

*Some statements in the manuscript require much stronger evidence than presented. For example "We downsampled the sequencing depth and found 2.8M reads per sample was enough in this experiment". Sequencing is far too complicated for such a statement to be made. Lowly expressed differentially expressed transcripts will be invisible at such a depth, and so there is always a trade-off between higher replicates benefitting the precision of estimation for highly expressed transcripts, and higher sequencing depth benefitting the ability to observe lowly expressed transcripts.*

We have revised the statements accordingly. This time we doubled the sequencing depth, and observe near saturation for the 18K genes detected in this experiment. However, we agree with the reviewer’s opinion, depending on the purposes of the research, requirements of sequencing depth might vary. Thus, we changed the statement, emphasizing the depth should be determined according to the projects.

*Why was the specificity fixed to 98.9%? This number seems arbitrary. The caption says that the specificity was improved, while also claiming it was fixed.*

Yes, it is completely arbitrary. Initially in the pilot experiment, we fixed the adjusted *p*-value and the corresponding specificity was happened to be 98.9%. Then in the following experiments, we kept using this number. The number indeed looks bizarre. Now we fixed the specificity to 95% and redid all the calculations. The conclusions won’t change by different specificity numbers. We are sorry for the typo in the figure legend, and we have fixed it.

*Why were the top 300 genes selected, instead of choosing a q-value or adjusted pvalue threshold? How were these genes selected? How were the 25 genes chosen for validation selected?*

We think a threshold of either adjusted *p*-value or top n will be an arbitrary number.

In our lab, we are trying to identify novel genes critical for germ cell development, and 300 genes is a number that we are comfortable to start with. That is why we ranked the list by adjusted *p*-values, then selected the top 300 genes to start with. The adjusted *p*-value of the 300th gene is 0.0058. The further 25 genes are selected based on their expression level, gene structure, and functional clues for further functional study, including genetic knockout. We have modified the main text accordingly.

In summary, we experimentally compared Decode-seq and BRB-seq thoroughly, and added new data and descriptions to the manuscript. We also fully agreed with the reviewer’s comments and modified the text accordingly. We hope these revisions and responses could address all issues raised by Reviewer #2.

**2^nd^ Round of review:**

**Reviewer 2**

The authors have addressed my previous concerns, in particular implementing BRB-seq in their lab and making comparisons of the two methods in Fig 1d and 1e, and in Supp Fig 6 and 7. In particular, as Decode-seq seems to offer an alternative strategy to BRB-seq (5' based vs 3' based), it may be an attractive alternative for researchers.

I have a few minor comments:

The correlation boxplot in Fig 1d is useful for an overview, but I would have preferred to seem some scatterplots of log counts for some pairs of replicates, both for Decode-seq and BRB-seq. The plots of log counts are useful for understanding what may induce lower or higher correlations.

Fig 1e is probably better presented as a simple bar chart. Specificity is set to 95% so it can be dropped here.

**Reviewer 3:**

In response to previous reviewer comments, the authors have now carried out a comparison to the published BRB-seq method. They have pointed out the key advantages of Decode-seq over BRB-seq as requested by other reviewers, which is primarily that BRB-seq either needs a run of an entire flow cell (which is more expensive) or an entire lane with a custom primer and no PhiX spike-in (which gives poorer read qualities because of base diversity issues). As they mention in their response letter in the current manuscript, the original BRB-seq paper used an entire flow cell and generated high-quality data. However, in the current manuscript they chose to use the second approach when they did BRB-seq, which results in slightly worse quality. Thus, their comparison of Decode-seq to BRB-seq is somewhat biased in their own favor in terms of quality, which is what they show in the paper.

To be fair they should be very clear that they are making this choice for BRB-seq and acknowledge that their comparisons in Fig. 1d, e and Supplementary Figures 6 and 7, which show that Decode-seq looks better, is due to this choice. It is not necessarily reflective of the optimal quality of BRB-seq data.

Second, they also use their own computational pipeline to analyze BRB-seq data, rather than the pipeline developed by the BRB-seq researchers (BRBseq Tools). It is possible that some of the differences are due to the pipelines which might be best tailored for each method.

Finally, they fail to mention one shortcoming of the Decode-seq approach which is that because it is 5' anchored and uses a template-switching oligo, it is biased towards full-length cDNA molecules, which may not be fully representative of the original pool of starting mRNA molecules. This needs to be addressed.

**Response to reviewers**

Thank you very much for your help in handling our manuscript of Decode-seq. We have carefully

read all comments from both reviewers, fully addressed all issues raised, and finished the revised

manuscript. All changes were highlighted in yellow. In general, we appreciate these two reviewer’s professional suggestions and critical comments, which helped us to improve this manuscript. The following are the point-by-point responses to the comments.

***Reviewer #2:***

*The authors have addressed my previous concerns, in particular implementing*

*BRB-seq in their lab and making comparisons of the two methods in Fig 1d and 1e, and in Supp*

*Fig 6 and 7. In particular, as Decode-seq seems to offer an alternative strategy to BRB-seq (5'*

*based vs 3' based), it may be an attractive alternative for researchers.*

We appreciate the reviewer’s recognition. We added this sentence in the last paragraph (page 4) to suggest that researchers could choose either Decode-seq or BRB-seq depending on their different conditions.

*I have a few minor comments: The correlation boxplot in Fig 1d is useful for an overview, but I*

*would have preferred to seem some scatterplots of log counts for some pairs of replicates, both*

*for Decode-seq and BRB-seq. The plots of log counts are useful for understanding what may*

*induce lower or higher correlations.*

We plotted three pairs of mean correlation and three pairs of the worst correlation for each group, and added as Supplementary Figure 7 (page 3).

*Fig 1e is probably better presented as a simple bar chart. Specificity is set to 95% so it can be*

*dropped here.*

We modified Figure 1e as suggested. Now it looks easier to interpret. Thanks!

***Reviewer #3:***

*In response to previous reviewer comments, the authors have now carried out a comparison to the published BRB-seq method. They have pointed out the key advantages of Decode-seq over BRB-seq as requested by other reviewers, which is primarily that BRB-seq either needs a run of an entire flow cell (which is more expensive) or an entire lane with a custom primer and no PhiX spike-in (which gives poorer read qualities because of base diversity issues). As they mention in their response letter in the current manuscript, the original BRB-seq paper used an entire flow cell and generated high-quality data. However, in the current manuscript they chose to use the second approach when they did BRB-seq, which results in slightly worse quality. Thus, their comparison of Decode-seq to BRB-seq is somewhat biased in their own favor in terms of quality, which is what they show in the paper. To be fair they should be very clear that they are making this choice for BRB-seq and acknowledge that their comparisons in Fig. 1d, e and Supplementary Figures 6 and 7, which show that Decode-seq looks better, is due to this choice. It is not necessarily reflective of the optimal quality of BRB-seq data.*

We appreciate the reviewer’s critical comment. In our introduction part (page 2), we had explained that the sequencing strategy used in the BRB-seq paper is not practical for many labs; therefore, at the beginning of the paragraph describing the comparison (page 3), we stated that we used the second strategy. And at the end of this paragraph, we had also given the premise “with standard sequencing settings” before drawing our conclusion. To be more clear as the reviewer suggested, we added several sentences at the beginning to emphasize again that the BRB-seq study used a customized sequencing strategy to generate high-quality data, which might be too expensive for many researchers. Thus, we chose the second strategy in the comparison. Hopefully this will address the reviewer’s concern.

We want to explain this point further. Like many other labs, we perform sequencing using commercial services or internal facilities with certain cost. The price here is about $1000 per lane

and $8000 per FC. In our study, we routinely sequenced Decode-seq libraries with only 10% lane

($100) for the QC purpose, as they can be mixed with other standard Illumina libraries. For BRBseq libraries, there is no easy way to perform this small scale pilot sequencing. Then, we fully

sequenced one library containing 30 samples, which requires us $1000 per library or $33 per

sample. However, to obtain the optimal quality as the BRB-seq paper showed, users have to devote the entire FC, which costs $8000. If the user has only one library containing 3o samples, then the cost is $267 per sample. If users could gather 8 libraries/240 samples, then the sequencing cost could be cut to $33. Apparently, it is not easy to gather 240 samples to be sequenced each time, and it is very risky to sequence at this scale without pilot QC sequencing. The above calculation varies in different labs, but the conclusion likely remains the same. The non-standard structure makes sequencing of BRB-seq libraries difficult for many small labs. High-quality data showed in the BRB-seq paper come at high cost.

Therefore, we are not biasing towards our own favor; we are showing how two methods perform

in a realistic scenario. It is fair for all readers if we clearly describe all the pros and cons of each

method.

*Second, they also use their own computational pipeline to analyze BRB-seq data, rather than the*

*pipeline developed by the BRB-seq researchers (BRBseq Tools). It is possible that some of the*

*differences are due to the pipelines which might be best tailored for each method.*

The reason is that we are not able to run BRBseq Tools which stopped working with an error:

Exception in thread "main" java.lang.NullPointerException

at DGEMatrixManager.createOutputDGE(DGEMatrixManager.java:135)

at BRBseqTools.main(BRBseqTools.java:44)

It seemed to be related with the combined human/mouse genomes, as the pipeline worked well

with a single genome. We are not Java programmers, and we cannot fix the problem. Our

computational pipeline was written in Python script language, which has only 3 files and less than 400 lines of code in total. We think it is easier to use and debug. So we chose to use our pipeline to process both datasets. The differences of read quality and mapping rates have been evident before the pipeline processing. Therefore, we don’t think using different pipelines made a major contribution to the performance difference.

*Finally, they fail to mention one shortcoming of the Decode-seq approach which is that because*

*it is 5' anchored and uses a template-switching oligo, it is biased towards full-length cDNA*

*molecules, which may not be fully representative of the original pool of starting mRNA molecules.*

*This needs to be addressed.*

We acknowledge the reviewer’s criticism. We added some sentences at the end of the comparison

paragraph (page 3), and also in the last paragraph (page 4). As discussed above, it is fair for all

readers if we can clearly describe the pros and cons of both methods. Depending on the different

situations, researchers could choose the appropriate method suite for their studies.

We genuinely appreciate both reviewers for the critical comments, which made the manuscript

more clear and comprehensive. We hope that our revision and explanations addressed the

reviewers’ concerns.
